# Response to “Refining FIB-4-based risk stratification in adverse drug reactions”

**DOI:** 10.1007/s00228-026-04101-y

**Published:** 2026-06-17

**Authors:** Joana Henze, Lennard Goette, Harald Dormann, Carolin V. Schneider, Corinna Meeßen, Anja Knüppel-Ruppert, Andrea Kriegisch-Stumpf, Matthias Schwab, Julia C. Stingl, Katja S. Just

**Affiliations:** 1 Institute of Clinical Pharmacology, University Hospital RWTH Aachen, Wendlingweg 2, D-52074 Aachen, Germany; 2 Emergency Department, Klinikum Fürth, Fürth, Germany; 3 Department of Medicine III, University Hospital RWTH Aachen, Aachen, Germany; 4 Central Emergency Department, Hospital Ingolstadt, Ingolstadt, Germany; 5 Dr. Margarete Fischer-Bosch-Institute of Clinical Pharmacology, Stuttgart, Germany; 6 Department of Clinical Pharmacology, and Pharmacy and Biochemistry, University Tübingen, Tübingen, Germany; 7 Department of Clinical Pharmacology and Pharmacoepidemiology, Heidelberg University Hospital, Heidelberg, Germany

Dear Editor,

We thank the authors for their thoughtful and constructive comments regarding our study on the association between elevated FIB-4 scores and serious outcomes among patients admitted with adverse drug reactions (ADRs) [1, 2].

We agree that interpretation of FIB-4 in the acute ADR setting requires caution, as transaminases and platelet counts may be influenced by acute illness, ADR-related organ injury, or systemic inflammation. Our study, however, aimed to evaluate FIB-4 primarily as a pragmatic and readily available clinical marker associated with ADR outcomes in real-world emergency care rather than as a definitive surrogate marker of histologically confirmed liver fibrosis. In this context, elevated FIB-4 may reflect chronic hepatic vulnerability, frailty, acute physiological stress, or a combination of these factors contributing to serious outcomes.

In response to the comments raised [1], we performed additional analyses adjusting for comorbidity burden using the Charlson Comorbidity Index. Although effect estimates became slightly less pronounced compared with the originally published analyses, associations between high fibrosis risk and both serious ADRs and poor discharge status remained clearly significant (Full cohort, Table 1). These findings suggest that the observed associations are not solely explained by multimorbidity.

We additionally conducted sensitivity analyses excluding ADR cases with hepatobiliary and/or hematologic manifestations defined by the respective MedDRA system organ classes. Excluding hepatobiliary ADR cases resulted in a slight attenuation of the association between high fibrosis risk and serious ADRs. However, across the remaining sensitivity analyses, effect estimates for serious ADRs and poor discharge status remained robust and in part became even more pronounced (Table 1). Associations with urgent triage remained non-significant throughout all analyses. Taken together, these findings argue against the interpretation that the observed associations are primarily driven by ADR-related alterations of FIB-4 components alone.

**Table 1:** Effect estimates for associations of outcomes of adverse drug reactions (ADRs) with high liver fibrosis risk including sensitivity analyses

	Urgent Triage, OR (95% CI)	Serious ADR, OR (95% CI)	Poor discharge status, OR (95% CI)
Full cohort (N=1087)	1.00 (0.74 – 1.34)	2.27 (1.48 – 3.48)	3.22 (2.02 – 5.14)
Sensitivity analyses			
Excluding cases with hepatobiliary ADR symptoms (n=1079)	1.02 (0.75 – 1.37)	2.20 (1.43 – 3.39)	3.31 (2.07 – 5.27)
Excluding cases with hematologic ADR symptoms (n=975)	0.91 (0.66 – 1.25)	2.40 (1.54 – 3.74)	3.65 (2.25 – 5.92)
Excluding cases with hepatobiliary and hematologic ADR symptoms (n=967)	0.93 (0.68 – 1.28)	2.33 (1.49 – 3.65)	3.76 (2.32 – 6.10)

Analyses adjusted for Charlson Comorbidity Index, male sex, and number of drugs

Urgent triage defined as red (immediate) and orange (very urgent); serious ADR defined as a stay in the intensive care unit, a life-threatening ADR and death; poor discharge status defined as permanent or fatal damage; high-fibrosis risk defined as FIB-4 score ≥2.67; OR: Odds Ratio; CI: confidence interval

We also agree that pharmacological heterogeneity deserves further investigation, as chronic liver disease may plausibly increase susceptibility to ADRs through structural and functional alterations in drug metabolism and transport as well as through a higher likelihood of clinically relevant drug-drug interactions [3]. However, only a relatively small proportion of ADR cases in our dataset presented with hepatobiliary manifestations, and we observed no differences in liver-specific admission diagnoses that would indicate frequent severe acute hepatic injury at the time of laboratory assessment. Moreover, serious ADR outcomes also occurred among patients with low FIB-4 scores, arguing against a simple reflection of ADR seriousness alone [2]. While future drug-analyses stratified by metabolic pathways, hepatotoxic potential, or interaction burden may provide additional mechanistic insight, our findings may rather support the hypothesis that elevated fibrosis risk itself represents a marker of increased vulnerability to unfavorable ADR outcomes independent of classical hepatotoxic drug effects. Interpretation of individual drug-specific effects is additionally complicated by the pronounced polypharmacy context within the cohort. Future longitudinal studies assessing FIB-4 before, during, and after ADR occurrence may help to better distinguish pre-existing fibrosis-related vulnerability from acute ADR-related laboratory alterations and their dynamic changes over time.

Overall, we appreciate these valuable comments, which helped refine the interpretation of our findings and further support the clinical relevance of FIB-4 as an easily accessible marker associated with serious outcomes among patients presenting with ADRs in emergency care settings.

## Data Availability

The analysed dataset can be accessed upon reasonable request to the corresponding author.

## Declarations

### Clinical trial registration

Registration was carried out via the German Clinical Trials Registry (DRKS-ID: DRKS00008979).
